# LRET-based distance measurements in the mammalian glutamate transporter EAAT3

**DOI:** 10.1186/2050-6511-13-S1-A52

**Published:** 2012-09-17

**Authors:** Kusumika Saha, SanthoshKannan Venkatesan, Azmat Sohail, Thomas Stockner, Simon Bulling, Gerhard F Ecker, Harald H Sitte, Walter Sandtner

**Affiliations:** 1 Institute of Pharmacology, Center for Physiology and Pharmacology, Medical University Vienna, 1090 Vienna, Austria; 2 Pharmacoinformatics Research Group, Department of Medicinal Chemistry, University of Vienna, 1090 Vienna, Austria

## Background

EAAT3 (excitatory amino acid transporter 3) mediates the regulation of synaptic transmission by reuptake of glutamate in the synaptic cleft. It is distributed in neuronal membranes and is selectively enriched in the neurons of the hippocampus, cerebellum and the basal ganglia. It belongs to the family of soluble carrier family 1 member 1 (SLC1A1) and is expressed in kidney, a wide variety of epithelial tissues, brain and eyes.

## Methods

The project utilizes the high-resolution crystal structure of GltPh, the bacterial orthologue to mammalian glutamate transporters. GltPh provides a structural framework for the determination of the helical movement in EAAT3. The structural rearrangement of the protein is caused by the helical movements which will be assessed by distance measurements using the technique of lanthanide resonance energy transfer (LRET). The protein will be expressed in *Xenopus laevis* oocytes. Lanthanide binding tags (LBT) will be inserted into the protein to chelate the lanthanide terbium which serves as the donor element. Cysteines will be reacted to an acceptor dye (bodipy FL).

## Expected results

The measured distances will allow us to obtain new insights into the structure-function relationship of the glutamate transporters which can be further investigated using different substrates and inhibitors.

## Discussion

The results obtained in this project will allow us to better understand pathophysiological conditions associated with mutations in EAAT3, for instance mutations causing human dicarboxylic aminoaciduria.

